# Focused Information Criterion for Restricted Mean Survival Times: Non-Parametric or Parametric Estimators

**DOI:** 10.3390/e24050713

**Published:** 2022-05-16

**Authors:** Szilárd Nemes, Andreas Gustavsson, Alexandra Jauhiainen

**Affiliations:** BioPharma Early Biometrics and Statistical Innovation, Data Science & AI, BioPharmaceuticals R&D, AstraZeneca, 43183 Gothenburg, Sweden

**Keywords:** parametric, non-parametric, information theory, model selection, survival analysis

## Abstract

Restricted Mean Survival Time (RMST), the average time without an event of interest until a specific time point, is a model-free, easy to interpret statistic. The heavy reliance on non-parametric or semi-parametric methods in the survival analysis has drawn criticism, due to the loss of efficacy compared to parametric methods. This assumes that the parametric family used is the true one, otherwise the gain in efficacy might be lost to interpretability problems due to bias. The Focused Information Criterion (FIC) considers the trade-off between bias and variance and offers an objective framework for the selection of the optimal non-parametric or parametric estimator for scalar statistics. Herein, we present the FIC framework for the selection of the RMST estimator with the best bias-variance trade-off. The aim is not to identify the true underling distribution that generated the data, but to identify families of distributions that best approximate this process. Through simulation studies and theoretical reasoning, we highlight the effect of censoring on the performance of FIC. Applicability is illustrated with a real life example. Censoring has a non-linear effect on FICs performance that can be traced back to the asymptotic relative efficiency of the estimators. FICs performance is sample size dependent; however, with censoring percentages common in practical applications FIC selects the true model at a nominal probability (0.843) even with small or moderate sample sizes.

## 1. Introduction

Restricted Mean Survival Time (*RMST*), the average survival time up to a given time point, is hailed as a model-free statistic, which is easy to interpret causally when summarizing survival data [1]. RMST has observed a resurgence in practical applications as an alternative to classical analysis based on log-rank tests or Proportional Hazard (PH) models when assessing between-group differences in survival analysis [2,3]. For clinical trial planning, the power of different analysis methods needs to be considered. There are indications that log-rank or PH tests generally have higher statistical power than RMST; however, this depends on the setting [4,5]. When estimated non-parametrically, RMST is less efficient than hazard-based methods estimated via semi- or fully parametric models under the proportional hazards assumption [6].

The heavy reliance on non-parametric or semi-parametric methods in a survival analysis has drawn some criticism [7,8]; however, as Meier and collaborators [9] point out, it is a rather challenging task to identify the correct parametric form for a certain problem. In addition, the censoring affects the efficacy of both parametric and non-parametric RMST estimators. Gardiner [10] used Kolmogorov–Smirnov, Andersen-Darling and Cramér-von Mises statistics to assess the goodness-of-fit of parametric distributions against the empirical Kaplan–Meier alternative prior to estimating RMST. Nemes and collaborators [11] concluded in a simulation study that, under model miss-specification, the non-parametric RMST estimator has superior efficacy in terms of the mean squared error (MSE) compared to parametric alternatives. The authors also concluded that parametric estimators reduce type II error rates (i.e., increased statistical power) if the correct distribution is identified. The percentage of censoring and the choice of restriction time are acknowledged by the authors to directly affect the comparability of parametric and non-parametric estimators.

The MSE offers an objective way to compare estimators in simulation studies where the true value of a parameter of interest is known. However, the validity of MSE comparisons is limited in practical situations, as the bias generally is unknown and is difficult to estimate. Building upon the Focused Information Criterion (FIC) by Claeskens and Hjort [12], Jullum and Hjort [13] developed a framework for objective comparison and model selection among parametric and non-parametric models, and this latest development of FIC is at the core of our study. FIC does not attempt to assess the overall fit of candidate models to observed data. Instead, candidate models are ranked based on the estimated precision of a parameter of primary interest. This ‘focus’ parameter does not need to be a specified parameter of a distribution, but can be any scalar summary of the data. As RMST captures the survival patterns in a single scalar measure, FIC offers a feasible framework for model selection.

In this paper, building upon Claeskens and Hjort [12] and Jullum and Hjort [13], we aim to establish the FIC framework for the model selection for RMST. We describe the mathematical framework needed for implementation. Thereafter, we look at factors affecting the performance of FIC, such as censoring type and rate as well as sample size. In addition, as with a real-life application, we illustrate possible gains in efficacy by using the parametric RMST estimators suggested by FIC without compromising interpretability. We also provide an indicative discussion of the interplay between the maximum follow-up time and chosen restriction time.

## 2. Notation and Assumptions

### 2.1. Notation and Nomenclature

We assume that survival times $X_{1},\ldots ,X_{n}$ for subjects j=1,…,n are independently and identically distributed (iid), according to the cumulative distribution function F(x)=P(X≤x) and survival function of interest S(x)=1−F(x)=P(X>x). Similarly we assume $C_{1},\ldots ,C_{n}$ to be iid censoring times according to the distribution function G(c) and survival function 1−G(c). Thus, the actual observed time for subject *j* is $T_{j}=\min\left(X_{j},\;C_{j},\right)$. Additionally, we have $\delta_{j}=I\left\{X_{j}\leq C_{j})\right\}$ as an event indicator that takes a value of 1 if the event of interest takes place before or on the given censoring time, and 0 otherwise. We assume independence between failure and censoring times. We let $t_{\left(1\right)}\leq ...\leq t_{\left(n\right)}$ denote the ordered observed survival times and $\delta_{\left(1\right)},...,\delta_{\left(n\right)}$ their associated indicator values.

In estimating the survival function *S* from the observed censored data ${\left(t_{\left(i\right)},\delta_{\left(i\right)}\right)}_{i=1}^{N}$, scientific literature almost exclusively uses the Kaplan–Meier Product-Limit estimator [14], expressed as

(1) $$\begin{array}{c} {\hat{S}}_{KM}\left(t\right)=\prod_{T_{i}\leq t}\left[1-\frac{\delta_{i}}{Y\left(T_{i}\right)}\right]\; \end{array}$$

where Y(t) is the number at risk at time *t*. If we have information about F(x) and if it is a member of a parametric family of distributions with *p*-variate parameter vector θ, then the likelihood function for the sample ($T_{j}$, $\delta_{j}$), j=1,...,n is

(2) $$\begin{array}{c} L\left(\theta |T_{j},\delta_{j}\right)=\prod_{j=1}^{n}f{\left(T_{j};\theta \right)}^{\delta_{j}}{\left\{1-F(T_{j};\theta )\right\}}^{1-\delta_{j}}. \end{array}$$

Further, we denote the first and second derivatives of the log-likelihood function, $\log L(\theta |T_{j},\delta_{j})$, as $u(T_{j};\theta )$ and $I(T_{j};\theta )$. We also define the information matrix as

(3) $$\begin{array}{c} J=-E_{F}\left\{I(T_{j};\theta )\right\}\;\mathrm{and}\;K={\mathrm{Var}}_{F}\left\{u(T_{j};\theta )\right\} \end{array}$$

Generally, *K* is considered an inefficient estimator of the information matrix; however, it plays an important role when robustness is of concern. Under some regularity conditions (see Chap 6 in [15]) the maximum likelihood estimator of θ, ${\hat{\theta }}_{MLE}$, satisfies

(4) $$\begin{array}{c} \sqrt{n}\left({\hat{\theta }}_{MLE}-\theta_{0}\right)\overset{D}{\to }N_{p}\left\{0,\Sigma \right\}, \end{array}$$

where $\theta_{0}$ is the unique minimizer of the Kullback–Leiber divergence and the least false parameter value; $N_{p}$ is a mean zero *p*-variate normal distribution with covaraince matrix Σ. If the assumed parametric model is the true model then J=K and $\Sigma =J{\left(\theta \right)}^{-1}$. Below, the subscript np denotes the non-parametric estimator and pm the parametric estimator, while the subscript 0 corresponds to the least false or best approximate value, as the minimizer of the Kullback–Leibler distance from the true model to the approximated model.

### 2.2. Restricted Mean Survival Time

Kaplan and Meier [14] suggested estimating the mean survival time (μ) as

(5) $$\begin{array}{c} {\hat{\mu }}_{KM}=\int_{0}^{\infty }t\;dF_{n}\left(t\right), \end{array}$$

where $F_{n}$ is the empirical distribution function. However, this is rarely estimable due to censoring and instead attention is paid to the τ-restricted mean survival time ($\mu_{\tau }$)

(6) $$\begin{array}{c} {\hat{\mu }}_{KM,\tau }=\int_{0}^{\tau }t\;dF_{n}\left(t\right)=\int_{0}^{\tau }{\hat{S}}_{KM}\left(t\right)\;dt. \end{array}$$

This approach disregards any information after τ and technically, this counts as Type I censoring, as the analysis is restricted to the interval (0,τ].

Alternatively, based on the plug-in principle, we can use the maximum likelihood estimates to calculate $\mu_{\tau }$ with the assumed distribution function as

$$\begin{array}{c} \mu_{\tau }=\int_{0}^{\tau }S\left(t;{\hat{\theta }}_{MLE}\right)\;dt. \end{array}$$

As the Kaplan–Meier estimator has an infinite number of parameters, $\sigma_{np}^{2}>\sigma_{pm}^{2}$. However, this presumes that F(t) is correctly identified. If F(t) is incorrectly selected, then the maximum likelihood estimator is asymptotically biased, resulting in a inflated MSE. Trading-off bias against variance is a cornerstone of the FIC, described in the next section. In this setting, the non-parametric estimator is considered unbiased, thus

(7) $$\begin{array}{c} MSE_{np}=0^{2}+\frac{v_{np}}{n}. \end{array}$$

and the MSE for the parametric estimator is given by

(8) $$\begin{array}{c} MSE_{pm}=b^{2}+\frac{v_{pm}}{n}, \end{array}$$

where *b* is the bias of the estimator and *v* represents the variance.

## 3. Focused Information Criterion for *RMST*

We now aim to deduce the FIC for RMST. We look at properties of the non-parametric estimator $\int_{0}^{\tau }{\hat{S}}_{KM}\left(t\right)\;dt$ and a parametric alternative denoted by $\int_{0}^{\tau }{\hat{S}}_{pm}\left(t\right)\;dt$. As $n\overset{}{\to }\infty$ based on Jullum and Hjort [13], we note that

(9) $$\begin{array}{c} \left(\begin{array}{c} \sqrt{n}\left(\int_{0}^{\tau }{\hat{S}}_{KM}\left(t\right)\;dt-\int_{0}^{\tau }S\left(t\right)\;dt\right)\\ \sqrt{n}\left(\int_{0}^{\tau }{\hat{S}}_{pm}\left(t\right)\;dt-\int_{0}^{\tau }S_{0}\left(t\right)\;dt\right) \end{array}\right)\overset{D}{\to }\left(\left(\begin{array}{c} Z\\ c^{t}J^{-1}U \end{array}\right)\right)∼\;N\left(\left(\begin{array}{c} 0\\ 0 \end{array}\right),\left(\begin{array}{cc} v_{np} & v_{c}\\ v_{c} & v_{pm} \end{array}\right)\right). \end{array}$$

Here, (Z,U) are zero mean normal variables with dimensions 1 and *p*. Next, we need to establish estimators for the parameters in Equation (9). For the variance of the non-parametric RMST, the empirical analogue of ${\hat{v}}_{np}=n^{-1}\sum_{i=1}^{n}IF\left(T_{i},{\hat{F}}_{n}\right)$ is a natural choice. Here, IF is the influence function of a statistical functional T(F) given by

(10) $$\begin{array}{c} IF\left(x;T,F\right)=\lim_{\epsilon \to 0}\frac{\left\{T[\left(1-\epsilon \right)F+\epsilon \delta_{x}]-T\left(F\right)\right\}}{\epsilon }, \end{array}$$

if this limit exists. Reid [16] was first to provide IF for censored data, and for the restricted mean survival time. Building upon the representation of the cumulative hazard function as a functional of two subsurvival functions $S^{u}=P\left(X>t,\delta =1\right)$ and $S^{c}=P\left(X>t,\delta =0\right)$ by Peterson [17], Reid [16] gives

(11) $$\begin{array}{c} IF\left(T_{i},F_{n},S^{u},S^{c}\right)=\int_{0}^{\tau }S\left(t\right)\left\{\frac{1\{s\leq t\}}{\left(S^{u}+S^{c}\right)\left(s\right)}+\int_{0}^{\min(s,t)}\frac{dS^{u}}{{\left(S^{u}+S^{c}\right)}^{2}\left(u\right)}\right\}\;dt, \end{array}$$

with τ<∞ and S(τ)>0. This reduces to the well known Greenwood plug-in estimator, which, as Eaton and collaborators [5] demonstrated based on Monte Carlo simulations, is closest to empirical and asymptotic variances. The Greenwood estimator is given by

(12) $$\begin{array}{c} \hat{V}\left(\mu_{\tau }\right)=\sum_{t_{i}\leq t}{\left[\int_{t_{i}}^{\tau }{\hat{S}}_{KM}\left(t\right)\;dt\right]}^{2}\frac{\delta_{i}}{Y_{i}\left(T_{i}\right)\left(Y_{i}\left(T_{i}\right)-\delta_{i}\right)}. \end{array}$$

The variance of the parametric estimator is defined from a model-agnostic viewpoint. The influence function of ${\hat{\theta }}_{MLE}=MLE\left(F\right)$ is given by

$$IF\left(T,F\right)=\lim_{\epsilon \to 0}\left\{MLE\left(F_{\epsilon }\right)-MLE\left(F\right)\right\}=J{\left(\theta \right)}^{-1}K\left(\theta \right)J{\left(\theta \right)}^{-1}$$

with

(13) $$\begin{array}{c} J=-\frac{1}{n}\sum_{j=1}^{n}\left\{I(T;\theta )\right\}\;\mathrm{and}\;K=\frac{1}{n}\sum_{j=1}^{n}\left\{u\left(T;\theta \right)u{\left(T;\theta \right)}^{t}\right\}. \end{array}$$

With the delta-method, this gives

(14) $$\begin{array}{c} v_{pm}={\left\{\frac{\partial \mu (\hat{\theta })}{\partial \theta }\right\}}^{t}J{\left(\theta \right)}^{-1}K\left(\theta \right)J{\left(\theta \right)}^{-1}\left\{\frac{\partial \mu (\hat{\theta })}{\partial \theta }\right\}. \end{array}$$

For the co-variance

(15) $$\begin{array}{c} v_{c}=\frac{1}{n}{\left\{\frac{\partial \mu (\hat{\theta })}{\partial \theta }\right\}}^{t}J^{-1}\sum_{j=1}^{n}IF\left(T_{j},{\hat{F}}_{n}\right)u\left(T_{j};\hat{\theta }\right). \end{array}$$

In Equation (9) we made the claim that $\sqrt{n}\left(\int_{0}^{\tau }{\hat{S}}_{KM}\left(t\right)\;dt-\int_{0}^{\tau }S\left(t\right)\;dt\right)$ has a limit normal distribution with a mean zero of a certain variance, implicitly assuming that $\int_{0}^{\tau }{\hat{S}}_{KM}\left(t\right)\;dt$ is asymptotically unbiased.

Generally, non-parametric estimators are unbiased; however, this is not true for the Kaplan–Meier integrals [14]. Meier [18] specified that ${\hat{S}}_{KM}\left(t\right)$ is “nearly unbiased” at a rate of $e^{-Y(t)}$.

Gill [19] provided stronger bounds for the bias

(16) $$\begin{array}{c} -F\left(t\right)H^{n}\left(t\right)\leq S\left(t\right)-{\hat{S}}_{KM}\left(t\right)\leq 0 \end{array}$$

where $H^{n}\left(t\right)=P\left(Y\left(t\right)=0\right)$ is the probability that the at risk set is empty.

Mauro [20] demonstrated that $Bias\langle \int_{0}^{\tau }t\;dF_{n}\left(t\right)\rangle \leq 0$. Zhou [21] was first to provide a lower bound for the bias

(17) $$\begin{array}{c} -\int_{0}^{\tau }tH^{n}\left(t\right)F\left(dt\right)\leq \mathrm{Bias}\langle \int_{0}^{\tau }t\;dF_{n}\left(t\right)\rangle . \end{array}$$

Stute [22] provided and improved version of the lower bound of the bias in the form of

(18) $$\begin{array}{c} -\int_{0}^{\tau }tG\left(t\right)H^{n-1}\left(t\right)F\left(dt\right)\leq \mathrm{Bias}\langle \int_{0}^{\tau }t\;dF_{n}\left(t\right)\rangle . \end{array}$$

It is evident that if there is no censoring, the terms of the lower bound vanish, and as the bias is strictly negative, $\int_{0}^{\tau }x\;dF_{n}\left(x\right)$ is unbiased. This is expected as in this case $\int_{0}^{\tau }x\;dF_{n}\left(x\right)=n^{-1}\sum_{i}X_{i}$. However, it is also evident that when censoring is present, the Kaplan–Meier integral can have a non-negligible large sample size bias. Maximum bias is observed at $\tau_{H}=\inf\left\{t:H\left(t\right)=1\right\}$, the least upper bound of support for the distribution function of *T*. In real life applications $\tau \ll \tau_{H}$. Additionally, the bias is more evident when *G* has short tails compared to *F*. As a result $H^{n}\left(t\right)$, or $H^{n-1}\left(t\right)$ on the interval (0,τ] is negligible and we can assume that the bias of ${\hat{S}}_{KM}∼0$.

The parametric estimator is asymptotically unbiased and based on Equation (9) for the bias $\hat{b}$, we have

(19) $$\begin{array}{c} \sqrt{\left(n\right)}\left(\hat{b}-b\right)\overset{D}{\to }c^{t}J^{-1}U∼N\left(0,\kappa \right) \end{array}$$

where $\kappa =v_{pm}+v_{np}-2v_{c}$.

Although, $\hat{b}$ is an approximately unbiased estimator for *b*, typically $\hat{b^{2}}$ overestimates $b^{2}$ with $E_{F}\left\{\hat{b^{2}}\right\}=b^{2}+\kappa /n+o\left(n^{-1}\right)$. Jullum and Hjort [13] noted that it is theoretically possible that $b^{2}<\kappa /n$ and introduced the following correction $\max(0,{\hat{b}}^{2}-\hat{\kappa }/n)$ in order to truncate negative estimates (i.e., no bias) to zero.

After we have established the necessary estimators, we can confirm the FIC scores for the RMST$\mu_{\tau }$ as

(20) $$\begin{array}{cc} FIC_{np} & =\frac{{\hat{v}}_{np}}{n} \end{array}$$

(21) $$\begin{array}{cc} FIC_{pm} & =\max\left\{0,{\hat{b}}^{2}-\frac{\hat{\kappa }}{n}\right\}+\frac{{\hat{v}}_{pm}}{n} \end{array}$$

Clinical trials mainly aim to compare two (or more) treatment arms, e.g., to test the difference in restricted means survival times between two groups (denoted 1 and 2, below)

(22) $$\begin{array}{c} \Delta =\int_{0}^{\tau }S_{1}\left(t\right)\;dt-\int_{0}^{\tau }S_{2}\left(t\right)\;dt. \end{array}$$

If Δ is estimated based on non-parametric models, then

(23) $$\begin{array}{cc} FIC_{np}^{\Delta } & =\frac{{\hat{v}}_{1_{np}}}{n_{1}}+\frac{{\hat{v}}_{2_{np}}}{n_{2}}. \end{array}$$

while if we use parametric estimators then

(24) $$\begin{array}{cc} FIC_{pm}^{\Delta } & =\max\left\{0,{\left({\hat{b}}_{1}-{\hat{b}}_{2}\right)}^{2}-\frac{{\hat{\kappa }}_{1}}{n_{1}}-\frac{{\hat{\kappa }}_{2}}{n_{2}}\right\}+\frac{{\hat{v}}_{1_{pm}}}{n_{1}}+\frac{{\hat{v}}_{2_{pm}}}{n_{2}}. \end{array}$$

Naturally, a mix of distributions or a mix of parametric and non-parametric estimators is possible.

## 4. Operating Characteristics of *FIC* for *RMST*

Jullum and Hjort [13] (Corollary 1) provided the upper probability limit of FIC selecting the true parametric model over the non-parametric one ($\alpha_{n}$) as $Pr(\chi_{1}^{2}<2)=0.843$. Likely, $\alpha_{n}$ is influenced by several factors that limit the amount of information available in the data. In the following, we assess how censoring and sample size affect $\alpha_{n}$. In addition, we discuss how the choice of τ and the relationship between τ and maximum follow-up time ($t_{max}$) might affect FIC.

The characteristics of the variance estimators for RMST have direct implications on FIC. The Greenwood variance estimator (Equation (12)) is a sum of a sequence of overlapping squared areas from $t_{i}$ to τ weighed by the square of the coefficient of the variation of S(t) at $t_{i}$. As noted previously, $v_{np}\leq v_{pm}$. When we have Type I censoring, the support set for $v_{np}$ and $v_{pm}$ coincide. If $t_{max}>\tau$, then the domain of the non-parametric estimator is (0,τ], while for the parametric estimator it is $\left(0,t_{max}\right]$. The proportion of the total Fisher Information contained in the censored data is just the proportion of observations that are not censored [23], and given X⊥⊥C⊥⊥τ we have

(25) $$\begin{array}{c} J(T,\theta )=J(X,\theta )Pr(X<\tau \wedge C) \end{array}$$

where $Pr\left(X<\tau \wedge C\right)=\int_{0}^{\tau }F\left(x\right)g\left(x\right)\;dx$ and if $\tau <t_{max}$ then $J_{t_{max}}{\left(\theta \right)}^{-1}<J_{\tau }{\left(\theta \right)}^{-1}$. If the parametric model is correct, and follow-up is not restricted to (0,τ] (i.e., random censoring) with J(T,θ)=J(X,θ)Pr(X<C), FIC ought to select the true parametric estimator with higher probability.

Within a reasonable restriction time, we expect that $\alpha_{n}$ is directly affected by the percent of censored observations and sample size. Here, we consider a scenario where we assume that the maximum follow-up time is τ, mirroring a clinical trial with Type I censoring at τ. The actual observed time for subject *j* is $T_{j}=\min\left(X_{j},C_{j}\wedge \tau \right)$ and $\delta_{j}=I\left\{X_{j}\leq C_{j}\wedge \tau \right\}$, a mix of Type I and random censoring. We assume exponential survival times with λ=1/365 and Type I censoring at τ=365, and evaluate a series of random exponential censoring times with hazard γ=0.1/365,...,3/365 with increments of 0.029. This resulted in a minimum overall censoring of 36.7%, and a maximum censoring of 75%. For each γ, we simulated a data set with n=100 and estimated FIC for the non-parametric and for the exponential RMST. The simulations were repeated 1000 times. The aim was to assess the true positive rate of choosing between the (true) exponential RMST and the non-parametric alternative.

**Figure 1:**
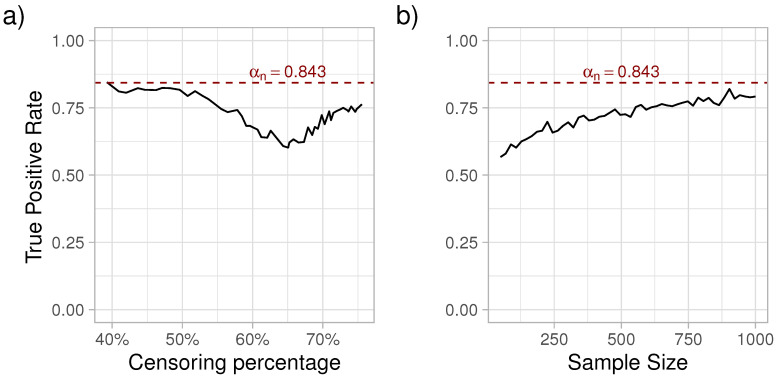
True positive rate of correctly identifying the exponential distribution as a function of censoring percentage (**a**) and sample size (**b**). The dashed horizontal line represents the theoretical limit ($\alpha_{n}=0.843$) of selecting the true parametric model over the non-parametric one.

As it can be observed in Figure 1, with increasing censoring, the sensitivity of FIC initially decreased, reaching a minimum at around 60% of censoring, followed by an increase in sensitivity. Next, with the censoring percentage at the point where the sensitivity was the lowest (γ=0.00448), we simulated survival data with varying sample sizes from 50 to 1000 subjects and estimated FIC. Each sample size was simulated 1000 times. As expected, the true positive rate of choosing the exponential distribution increased with the sample size. For a more detailed look at the patterns recorded in Figure 1, please see the Appendix A.

## 5. Practical Application

**Figure 2:**
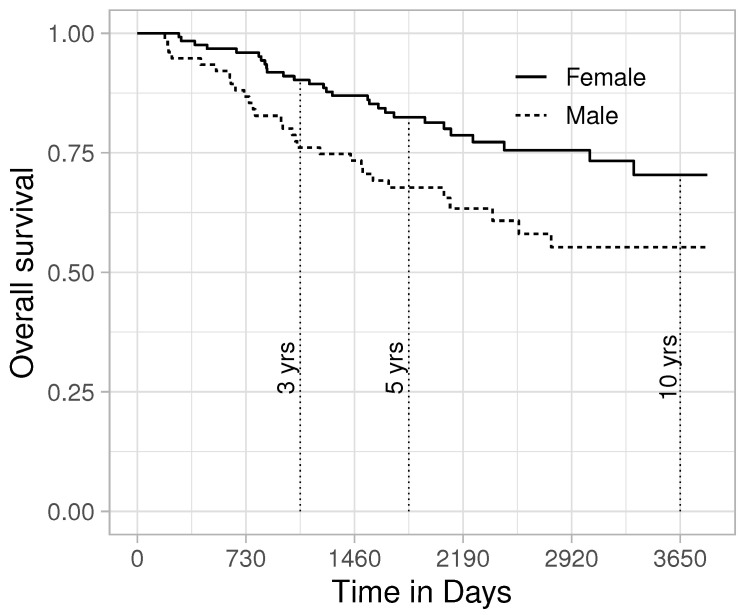
Overall survival after melanoma diagnosis.

The survival rate of melanoma has increased in recent decades, with approximately two-thirds of the patients surviving 5 years or more after diagnosis, with women generally having better survival than men [24]. Using a data set compiled by Drzewiecki and collaborators [25], we will assess the possibility to improve the efficiency of an RMST analysis of sex-specific survival. Data from 126 female and 79 male melanoma patients are included in the analysis (data can be found in the “timereg” R package). As can be observed in Figure 2, females have better survival prospects than males. Next, we analyse whether the restricted mean survival time at 3, 5 and 10 years differ between the sexes. As competing models, we consider the non-parametric estimator and the Exponential, Weibull, Gamma, Generalized Gamma and Log-logistic distributions. The combination of the Exponential distribution for men and Gamma distribution for women was flagged by FIC as a better alternative than the purely non-parametric estimators (Table 1).

On average, women had 65 days longer survival in the initial 3 years. The bias of the parametric estimator was negligible (0.7 days). Additionally, the parametric model reduced the 95 % confidence interval (CI) length with 27.5%, a considerable gain.

On average, women had 165 days longer survival in the initial 5 years. Just as for the 3-year survival, the Exponential-Gamma combination best described the data at 5 years, and reduced the 95% CI length with 18%. However, it should be noted that the bias was 14 days, which is an 8.5% bias. At 10 years, the RMST difference between men and women increased to 15.5 months. Still, parametric estimation increased the efficiency; however, the reduction in CI length was less than 1%, a very minor gain compared to the non-parametric estimator.

## 6. Discussions

In this paper, we have introduced the FIC [12,13] as a tool for the model selection for RMST. While FIC has a well established theory and is applicable in a wide range of areas, using FIC as a tool for selection of the best RMST model has some characteristics that need to be considered. First, we need to consider that the non-parametric RMST estimator is not consistent and is biased. Likely, this will have minor implications in practical applications; nevertheless, researchers should consider this aspect. If the at risk set at the chosen restriction time τ is the empty set or contains very few participants, the bias can be non-negligible. Second, the censoring percentage and type of censoring (type II or random, possibly hybrid) affects the efficiency of parametric and non-parametric estimators differently. Third, the variance of the Kaplan–Meier survival curve at any time *t* is based on information up to t−. The parametric survival curve estimator use information up to τ in the case of Type I censoring, or $t_{max}$ in the case of random censoring.

Jullum and Hjort [13] concluded that the upper probability limit of FIC selecting the true parametric model over the non-parametric one ($\alpha_{n}$) to be $Pr(\chi_{1}^{2}<2)=0.843$, a probability that was replicated in our simulations. This probability was obtained when the exponential estimator was tested against the non-parametric estimator in a setting when the exponential model was the true one and the censoring was due the restriction at τ. We observed that $\alpha_{n}$ was dependent on the censoring percentage. In addition, reaching $\alpha_{n}=0.843$ is sample size dependent.

In clinical trials of chronic diseases, τ coincides with the end of the follow-up. In observational studies, often $\tau \ll t_{max}$, thus the information contained in $\left(\tau ,t_{max}\right]$ might offer an extra advantage for the parametric variance estimator. However, the same information in $\left(\tau ,t_{max}\right]$ might bias the parametric survival estimate up to τ and induce bias in ${\hat{\mu }}_{\tau }$. This is more apparent when outliers are present, which usually appear on the right tail of the distribution. This depends on the assumed distribution, as Aranda [26] highlighted, where, e.g., exponential survival curves are less affected than Weibull survival curves.

As illustrated by the analysis of the melanoma data, FIC selected the best fitted model that minimizes MSE. However, just as Akaike or the Bayesian Information Criterion (AIC and BIC), it offers a ranking of competing models, but not a direct gauge of model fit or quality. At the 10-year restriction time, the parametric estimator was ranked first, but the statistical gains (i.e., lower MSE) of choosing the parametric estimator was negligible. Only looking at FIC ranks is likely not enough, but one should consider the distance between the competing models on the FIC scale. Just as with AIC and BIC, this requires further research.

One practical difficulty of parametric estimation of the RMST lies in the selection of parametric distribution(s). A set of competing parametric families can be selected based on subject-specific disease knowledge and by graphical examination of the hazard. The aim should not be to identify the true underlying distribution that generated the data, but to identify families of distributions with similar shapes [27] and by simultaneously looking at the bias and variance with FIC to decide how much model miss-specification can be tolerated [28] in order to increase efficiency.

In conclusion, we advocate the adaptation of the FIC framework for model selection for RMST. Studies with relatively short restriction times (i.e., restriction time shorter than the mean/median survival time) can greatly benefit from moving from a non-parametric estimation to a parametric one. It is relatively easy to identify families of distributions with similar shapes as the observed data for shorter follow-times, which would decrease the bias. In observational studies where $t_{\max}>\tau$, we recommend a first analysis to be conducted so that the support set of both parametric and non-parametric estimators is (0,τ]. This setting will likely result in a smaller bias for the parametric RMST estimator and would aid interpretability. Naturally, as FIC trades off bias against variance, a reduced variance might outweigh the bias of the parametric estimator on $\left(0,t_{\max}\right]$. Yet another argument for restricting attention to (0,τ] is that the distribution that FIC selects might convey important medical/biological information.

## Figures and Tables

**Table 1:** Difference in RMST (Δ) and associated FIC for women compared to men expressed in days at 3, 5 and 10 years for melanoma patients estimated with non-parametric Kaplan–Meier integrals and a combination of Exponential and Gamma distributions.

Timepoint	Model	Δ (Days)	Bias of Δ	$\sqrt{\mathrm{FIC}}$	95% CI for Δ
3 yrs	Non-param.	65.32	-	30.02	6.47; 124.17
	Param.	64.54	−0.78	21.74	21.92; 107.17
5 yrs	Non-param.	165.19	-	67.10	36.66; 299.72
	Param.	153.92	−14.83	54.83	46.46; 261.40
10 yrs	Non-param.	468.19	-	183.06	109.40; 826.99
	Param.	452.62	−15.57	181.51	96.86; 808.37

**Figure A1:**
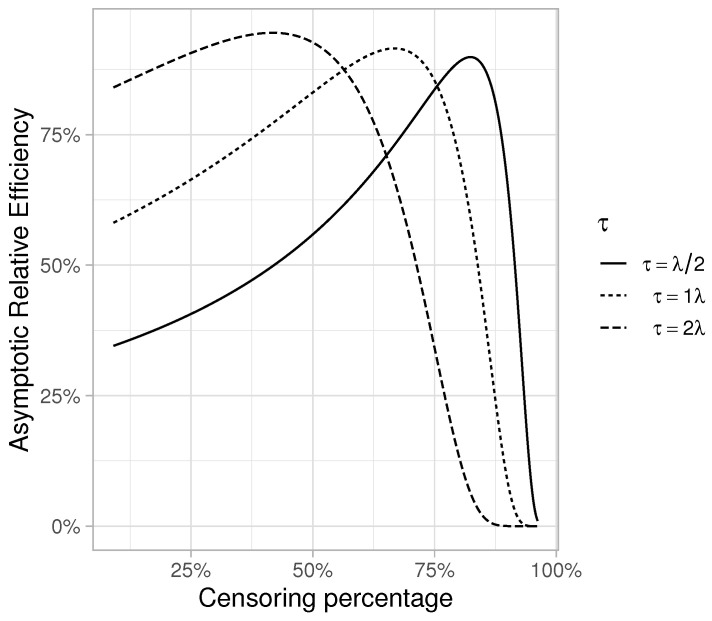
Asymptotic relative efficiency of the parametric and non-parametric variance estimators for RMST as a function of censoring percentage and restriction (τ) time.

## Data Availability

Not applicable.

## Abbreviations

The following abbreviations are used in this manuscript:

CI	Confidence Interval
FIC	Focused Information Criterion
IF	Influence Function
KM	Kaplan–Meier
MLE	Maximum Likelihood Estimator
np	Non-parametric
pm	Parametric
RMST	Restricted Mean Survival Time

## Appendix A. Behavior of FIC for RMST as a Function of Censoring

Figure 1a in the main text exhibited an interesting pattern, namely that the probability of selecting the true exponential RMST over the non-parametric estimator had a v-shaped curve, with $Pr_{F_{n}}\left(FIC_{pm}\leq FIC_{np}\right)$ being the lowest at around 65% of the data censored. Here, we will look at FIC and its components, to elucidate the mechanisms behind this pattern. As noted in the main text

(A1) $$\begin{array}{cc} FIC_{pm} & =\max\left\{0,{\hat{b}}^{2}-\frac{\hat{\kappa }}{n}\right\}+\frac{{\hat{v}}_{pm}}{n}. \end{array}$$

If the parametric model is the true data generating process then, at least asymptotically, $\hat{b}=0$, and in practical situations it is expected that $\hat{b}$ is close to zero and $FIC_{pm}$ is dominated by the variance. Please see Appendix B for more details.

Starting with the Greenwood variance estimator (Equation (12)) and then replacing each term by the asymptotic limiting counterparts, the non-parametric asymptotic variance (AVar) is

(A2) $$\begin{array}{c} AVar\left(\mu_{np}\right)=\int_{0}^{\tau }{\left\{\int_{x}^{\tau }S\left(t\right)\;dt\right\}}^{2}\frac{h(x)}{n(1-F(x))(1-G(x))}\;dx. \end{array}$$

Assuming exponential survival times with rate λ and exponential censoring times with rate γ then

(A3) $$\begin{array}{c} \frac{1}{n}AVar\left(\mu_{np}\right)=\int_{0}^{\tau }\frac{{\left(e^{-\lambda \tau }-e^{-x\lambda }\right)}^{2}}{\lambda e^{-x(\lambda +\gamma )}}\;dx. \end{array}$$

The variance of the parametric estimator when the assumed parametric model is the true model (and J=K) is given by

(A4) $$\begin{array}{c} AVar\left(\mu_{pm}\right)={\left\{\frac{e^{-\lambda \tau }\left(1+\lambda \tau \right)-1}{\lambda^{2}}\right\}}^{2}\frac{\lambda^{2}}{\sum_{i}\delta_{i}}. \end{array}$$

Here, $\sum_{i}\delta_{i}=nPr\left(X<C\right)=n\lambda {\left(\lambda +\gamma \right)}^{-1}$. Next, we need to establish the Asymptotic Relative Efficiency (ARE) between the non-parametric (Equation (A3)) and parametric variance (Equation (A4)) as

$$\begin{array}{c} ARE=\frac{AVar(\mu_{pm})}{AVar(\mu_{np})}. \end{array}$$

Without providing a closed form solution, we can observe that the sample size *n* is factored out. Thus, for a given τ the ARE is a function of the proportion of censored observations (Figure A1). The v-shaped curve of Figure 1 is present here as well. Miller [7] and Jullum and Hjort [8] have assessed the ARE of parametric and non-parametric variance estimators, concluding that the maximum ARE is 64%. As Figure A1 indicates, the maximum ARE is much higher; however, for realistic restriction times (≤mean survival time), this is achieved at a high censoring percentage, i.e., when the censoring distribution has shorter tails than the survival distribution. This might result in $t_{max}<\tau$, which complicates the estimation of $\int_{0}^{\tau }\hat{S}\left(t\right)\;dt$. Kaplan and Meier [14] did not define S(x) for $t_{max}<x$ and $\delta_{t_{max}}=0$. Efron [29] proposed a modification so that $\forall x>t_{max}$S(x)=0, while Gill [19] proposed $S\left(x\right)=S\left(t_{max}\right)|\forall x>t_{max}$. Both modifications would bias RMST and in light of guidelines by Eaton [5], RMST should not be calculated in these settings.

In closing, we can conclude that the v-shaped curve of the true positive rate of FIC choosing the true exponential distribution is due to the relation of the asymptotic relative efficiency of parametric and non-parametric estimators to censoring. This concludes the discussion of Figure 1a.

## Appendix B. Limit Probability as a Function of Censoring and Sample Size

Figure 1b in the main text illustrates, with the help of simulation, the convergence to the limiting probability that FIC would select a parametric model over the non-parametric one as a function of censoring and sample size. Using the notation and theory outlined in the appendix by Jullum and Hjort [13], we note that

$$\begin{array}{c} Pr_{F_{n}}\left(FIC_{pm}\leq FIC_{np}\right)\overset{}{\to }Pr\left\{\chi_{1}^{2}\left(\frac{n{\left({\hat{\mu }}_{np}-{\hat{\mu }}_{pm}\right)}^{2}}{v_{np}-v_{pm}}\right)\leq 2\right\}. \end{array}$$

Here $\chi_{1}^{2}\left(\zeta \right)$ is a non-central distributed variable with 1 degree of freedom and a non-centrality parameter ζ. If the considered parametric model is the true one and we have unbiased estimates, then ζ=0 and $Pr_{F_{n}}\left(FIC_{pm}\leq FIC_{np}\right)=0.843.$

As noted in the main text, ${\hat{\mu }}_{np}$ is downward biased and the bias may decrease to zero at a rate slower than $\sqrt{n}$ [30]. The simulation studies in Section 5 in the main text assumed exponential survival times. Maximum likelihood estimators are consistent; however, they can have a small sample bias. The bias of the maximum likelihood estimate of the rate parameter (λ) of the exponential distribution ${\left(n-1\right)}^{-1}\lambda$ rapidly decreases with increasing sample size. We note that β=E[X] is the expectation of the uncensored survival times; then, with the help of the delta-method, we can establish the bias of ${\hat{\mu }}_{pm}$ as

$$\begin{array}{c} b_{pm}=-\frac{1}{2n}\frac{\tau^{2}e^{-\tau \beta^{-1}}}{\beta } \end{array}$$

Just as for the non-parametric estimate, we have a downward bias. In addition, $b_{pm}\overset{}{\to }0$ as $n\overset{}{\to }\infty$ or $\tau \overset{}{\to }\infty$. Due to the bias of both parametric and non-parametric estimates and different convergence rates, we expect that ${\hat{\mu }}_{np}-{\hat{\mu }}_{pm}\neq 0$. We can conclude that if $n\overset{}{\to }\infty$ then $Pr_{F_{n}}\left(FIC_{pm}\leq FIC_{np}\right)\overset{}{\to }0.843$.

The simulation in Section 5 assumed hybrid random and Type I censoring. This assumes that no information is recorded after τ. In observational studies, the maximum follow-up usually exceeds τ. The asymptotic relative efficiency of the parametric estimator in the setting described in Section 5 with $T_{j}=\min\left(X_{j},C_{j}\wedge \tau \right)$ is around 87% of the estimator with $T_{j}=\min\left(X_{j},C_{j}\right)$. Thus, considering the available data after τ increases the convergence toward 0.843. This concludes the discussion of Figure 1b.
